# The Short-Term Exposure to SDHI Fungicides Boscalid and Bixafen Induces a Mitochondrial Dysfunction in Selective Human Cell Lines

**DOI:** 10.3390/molecules26195842

**Published:** 2021-09-26

**Authors:** Donatienne d’Hose, Pauline Isenborghs, Davide Brusa, Bénédicte F. Jordan, Bernard Gallez

**Affiliations:** 1 Biomedical Magnetic Resonance, Louvain Drug Research Institute (LDRI), Université catholique de Louvain (UCLouvain), 1200 Brussels, Belgium; donatienne.dhose@uclouvain.be (D.d.); pauline.isenborghs@gmail.com (P.I.); benedicte.jordan@uclouvain.be (B.F.J.); 2 CytoFlux-Flow Cytometry Platform, Institut de Recherche Expérimentale et Clinique, Université catholique de Louvain (UCLouvain), 1200 Brussels, Belgium; davide.brusa@uclouvain.be

**Keywords:** EPR, oxygen consumption rate (OCR), superoxide, mitochondria, SDHI, Boscalid, Bixafen

## Abstract

Fungicides are used to suppress the growth of fungi for crop protection. The most widely used fungicides are succinate dehydrogenase inhibitors (SDHIs) that act by blocking succinate dehydrogenase, the complex II of the mitochondrial electron transport chain. As recent reports suggested that SDHI-fungicides could not be selective for their fungi targets, we tested the mitochondrial function of human cells (Peripheral Blood Mononuclear Cells or PBMCs, HepG2 liver cells, and BJ-fibroblasts) after exposure for a short time to Boscalid and Bixafen, the two most used SDHIs. Electron Paramagnetic Resonance (EPR) spectroscopy was used to assess the oxygen consumption rate (OCR) and the level of mitochondrial superoxide radical. The OCR was significantly decreased in the three cell lines after exposure to both SDHIs. The level of mitochondrial superoxide increased in HepG2 after Boscalid and Bixafen exposure. In BJ-fibroblasts, mitochondrial superoxide was increased after Bixafen exposure, but not after Boscalid. No significant increase in mitochondrial superoxide was observed in PBMCs. Flow cytometry revealed an increase in the number of early apoptotic cells in HepG2 exposed to both SDHIs, but not in PBMCs and BJ-fibroblasts, results consistent with the high level of mitochondrial superoxide found in HepG2 cells after exposure. In conclusion, short-term exposure to Boscalid and Bixafen induces a mitochondrial dysfunction in human cells.

## 1. Introduction

**Figure 1:**
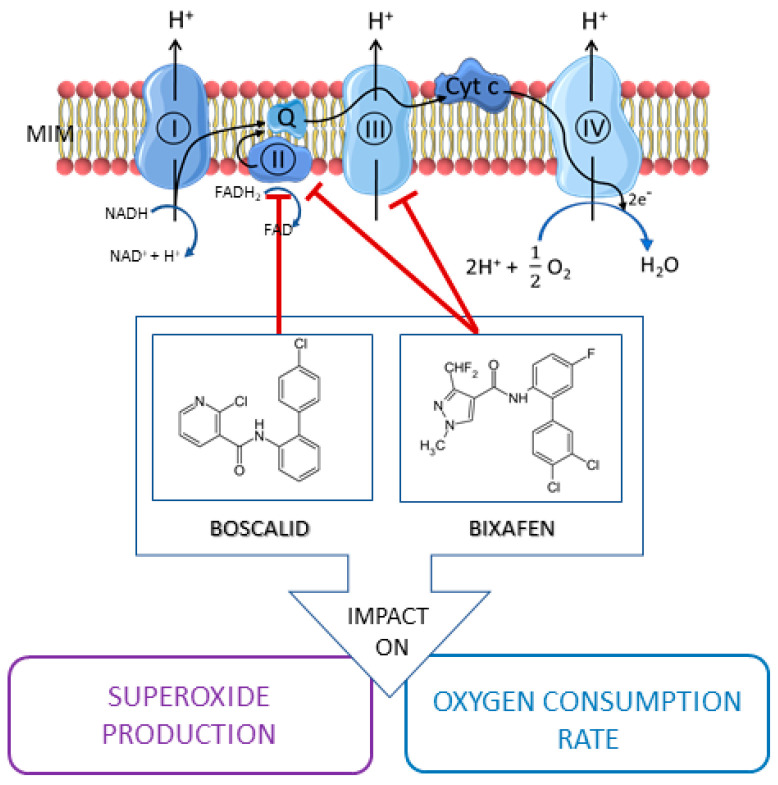
Effect of SDHI-fungicides on mitochondrial ETC. Boscalid and Bixafen block the complex II of the ETC, Bixafen also inhibiting complex III. By disrupting mitochondrial function, SDHIs may impact the production of mitochondrial superoxide radical and the oxygen consumption rate.

Fungicides are intensively used in agriculture to prevent or inhibit the growth of fungi causing plant damage. The most widely used fungicides are succinate dehydrogenase inhibitors (SDHIs) [1] that act by blocking succinate dehydrogenase (SDH), the complex II of the mitochondrial electron transport chain (ETC) (Figure 1). Of commercialized SDHIs, Bixafen and Boscalid account together for about 50% of the sales [2]. SDHIs are generally considered as safe by pesticide-manufacturing companies and safety agencies [1,2,3]. However, recent alarming reports have demonstrated that SDHIs used as fungicides were not selective for their fungi targets but also block the honeybee, earthworm and human SDH with IC50 values in the µM range [4]. This is likely due to the high degree of conservation of a large portion of the SDH sequences throughout different species [4]. The same authors also showed that pre-existing mitochondrial defects, such as partial SDH dysfunction, increased the susceptibility to SDHIs after several days of exposure [4].

Other recent reports revealed neurodevelopmental defects in zebrafish models after exposure of embryos to Bixafen or Boscalid a few hours post-fertilization [5,6] as well as severe cardiac toxicity [7]. Chronic exposures (28 days) of adult zebrafish led to alterations in carbohydrate and lipid metabolism, and induced damage in the kidneys and the liver [8]. A genotoxic effect was also shown in human cells after chronic exposure. The frequencies of micronuclei and binucleated cells with micronuclei were increased in cultured human lymphocytes treated with Boscalid for 72 h [9]. Using an assay based on histone γH2AX phosphorylation, genotoxicity was also observed after 24 h of Bixafen treatment in SH-SY5Y kidney cell line and in human lymphocyte Jurkat Tcells [10].

All the effects described earlier were obtained after chronic exposure to SDHIs. Benit et al. considered it was relevant to use µM range concentrations as the Acceptable Daily Intake (ADI) values, which should lead to comparable concentrations in blood after exposure [4]. They calculated that an ADI of 0.04 and 0.02 mg/kg/day, for Boscalid and Bixafen, respectively, should lead to a blood concentration of approximately 1 µM [4]. This was criticized by the European Crop Protection Association (ECPA, representing companies marketing pesticides) in a comment on Benit’s paper, considering that “*it does not account for the fact that SDHIs do not remain in the blood vessel compartment indefinitely but are rapidly distributed within the body*” [11]. We still believe that studying the effect in the µM range is appropriate. Indeed, it should be emphasized that the potential exposure limits for agricultural workers manipulating pesticides are much larger, as AOELs (Acceptable Operator Exposure Levels) for Boscalid and Bixafen are 0.1 mg/kg/day and 0.13 mg/kg/day, respectively. With the oversimplification of 100% of dose reaching the blood after absorption, for an operator with a body weight of 70 kg with 5 L of blood, it would lead to a peak concentration of 4.1 and 4.3 µM for Boscalid and Bixafen, respectively. Knowing that both compounds are readily absorbed after oral administration (83% and 56% for Bixafen and Boscalid, respectively) [12,13,14], and despite extensive metabolism and biliary excretion (elimination of radioactivity from radiolabeled Bixafen is characterized by a half-life in plasma of 8–9 h and a mean residence time of 13–19 h [12]), it is still reasonable to assume a theoretical short-term exposure of cells to µM concentration of SDHIs if exposed at levels close to the AOELs. The remaining issue to be resolved is to address the effect of short-term exposure to SDHIs of human cells exposed during their normal physiological functions.

To fill this gap, we tested the mitochondrial function of human cells exposed for a short time to Boscalid and Bixafen, the two most used SDHIs. For this purpose, we selected PBMCs (Peripheral Blood Mononuclear Cells), HepG2, and BJ human cell lines. Those cells are the most likely to be in contact with SDHIs after exposure: blood circulating cells and liver cells after oral intake (Bixafen and Boscalid accumulate in liver [12,13,14]), and fibroblasts after skin exposure. We focused on the mitochondrial ETC function of these cells. Interestingly, some SDH blockers (such as α-tocopheryl succinate, vitamin E-derived compounds linked to triphenylphosphonium group, malonate, 3-nitropropionate, lonidamine) have been evaluated in cancer therapy because the leakage of electrons to molecular oxygen leads to excessive production of superoxide ions and cancer cell apoptosis [15,16,17,18,19,20]. Hypothesizing that SDHIs-fungicides may present a similar mode of action, we used Electron Paramagnetic Resonance (EPR) spectroscopy which is particularly suitable to assess the oxygen consumption rate (OCR) [21,22,23] as well as the production of mitochondrial superoxide [23,24,25,26,27,28,29]. In addition, we evaluated the impact of short-term exposure to SDHIs on cell apoptosis and necrosis.

## 2. Results

### 2.1. Boscalid and Bixafen Decreased OCR in the Three Human Cell Lines 

**Figure 2:**
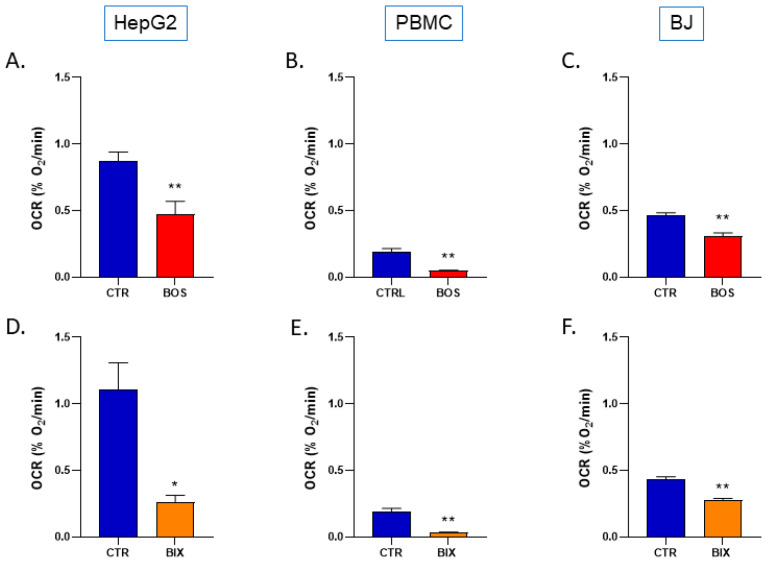
Oxygen consumption rate of control (DMSO 0.1%) and SDHIs-exposed human cells (1µM for 2 h). Top row: Boscalid-treated cells. (**A**): HepG2 liver cells, (**B**): PBMCs, (**C**): BJ-fibroblasts. Bottom row: Bixafen-treated cells. (**D**): HepG2 liver cells, (**E**): PBMCs, (**F**): BJ-fibroblasts. Bars represent mean ± SEM (%O_2_/min). N = 3, (*): *p* < 0.05, (**): *p* < 0.01.

The OCR was significantly decreased in cells that were exposed for 2 h to 1µM Boscalid and Bixafen (Figure 2). For HepG2, both Boscalid and Bixafen decreased the OCR in a significant manner with 46% and 76% inhibition, respectively, compared with the controls. The same results were observed in PBMCs where Boscalid and Bixafen inhibited OCR by 75% and 83%, respectively, and in BJ-fibroblasts with an inhibition of OCR by 33% and 36%, respectively.

**Figure 3:**
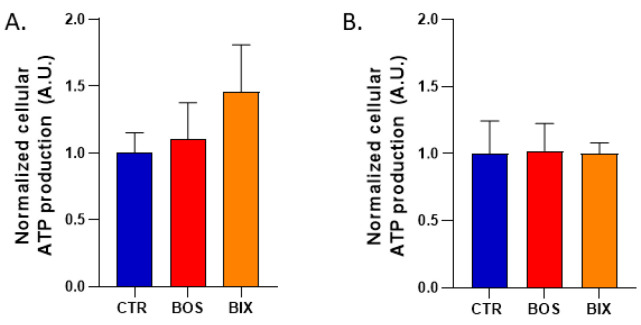
Cellular ATP production of HepG2 cells after treatment with DMSO 0.1% (CTR), Boscalid 1 µM (BOS) and Bixafen 1 µM (BIX) for 2 h (**A**) and 24 h (**B**). Data were normalized to controls. Bars represent mean ATP production ± SEM (A.U.), N = 3.

We further explored the metabolic consequences of the decrease in OCR in HepG2 cells. The change in the OCR observed did not lead to a change in the ATP level measured after 2 h or 24 h exposure to Boscalid and Bixafen (Figure 3).

### 2.2. Impact of SDHIs on Mitochondrial Superoxide Generation in Human Cell Lines

**Figure 4:**
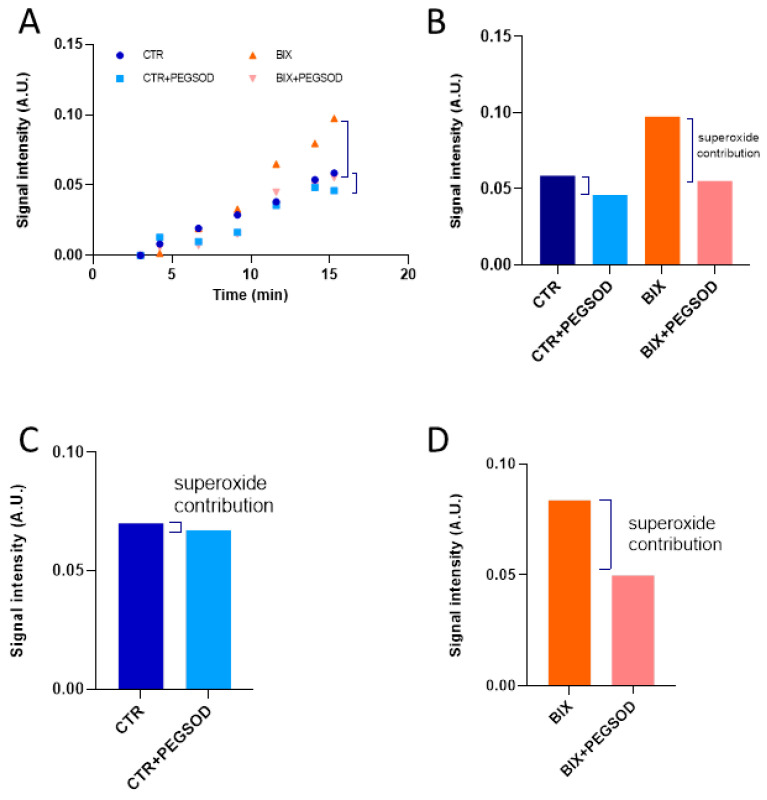
Illustration of the evaluation of mitochondrial superoxide assessment using EPR. Top row: typical experiment with exposure of HepG2 cells to Bixafen. (**A**): Time course evolution of EPR signal intensity of Mito-TEMPO^•^ produced from Mito-TEMPO-H. (**B**): EPR signal intensity recorded for control cells and exposed cells 15 min after the start of the EPR experiment. The incubation with PEG-SOD2 allowed the evaluation of the superoxide contribution to the oxidation of the mitochondrial sensor. Bottom row: Mean values of EPR signal intensity at time t = 15 min recorded for 3 independent experiments. (**C**): Control HepG2 cells. (**D**): HepG2 cells exposed for 2 h to Bixafen.

Mitochondrial superoxide levels were determined using EPR with Mito-TEMPO-H as a specific mitochondrial superoxide probe ± PEG-SOD2 to specifically assign signals to mitochondrial superoxide [28] (Figure 4).

**Figure 5:**
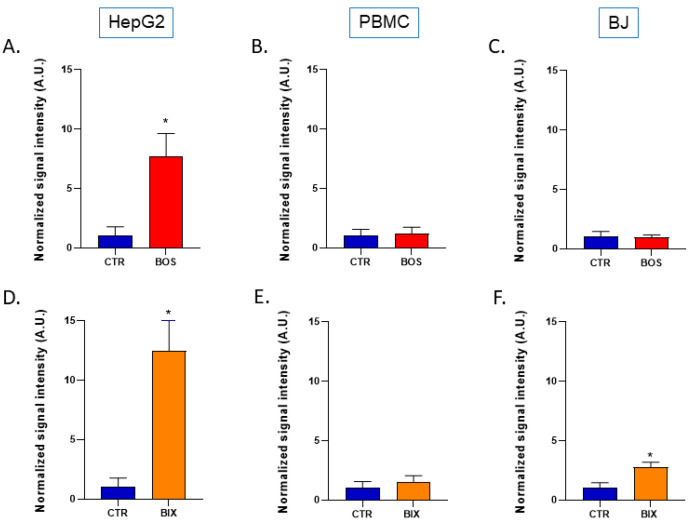
Level of mitochondrial superoxide in control (DMSO 0.1%) and SDHIs-exposed human cells (1 µM for 2 h). Signal intensity of Mito-TEMPO^•^ at time point 15 min (normalized to control). Top row: Boscalid-treated cells. (**A**): HepG2 liver cells, (**B**): PBMCs, (**C**): BJ-fibroblasts. Bottom row: Bixafen-treated cells. (**D**): HepG2 liver cells, (**E**): PBMCs, (**F**): BJ-fibroblasts. Bars represent mean ± SEM. N = 3, (*): *p* < 0.05.

**Figure 6:**
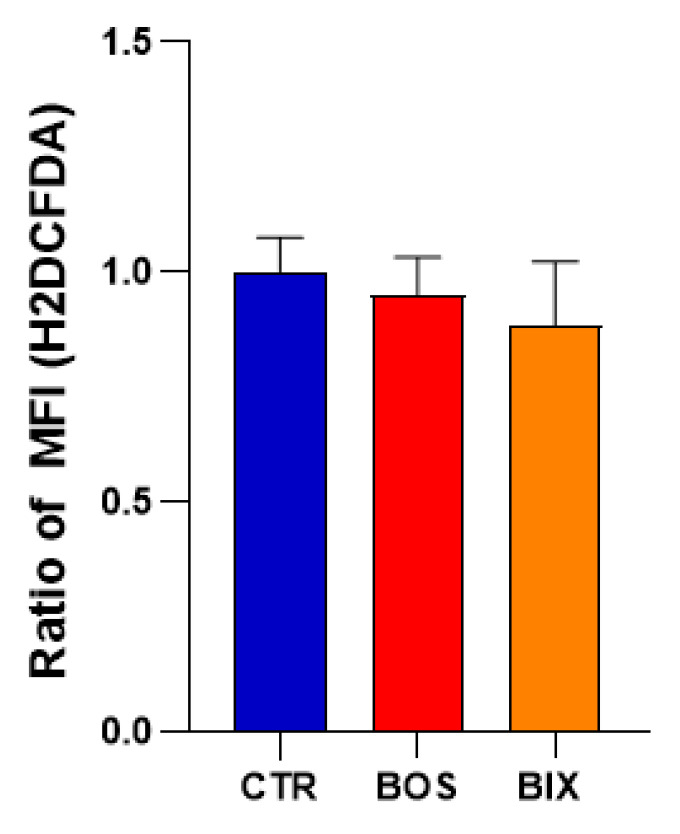
Total ROS production in HepG2 cells after treatment with DMSO 0.1% (CTR), 1 µM Boscalid (BOS) and 1 µM Bixafen (BIX) for 2 h, measured using H_2_DFCDA probe. Data were normalized to controls. Bars represent mean ratio of MFI (mean fluorescence intensity) ± SEM (A.U.), N = 3.

After 2 hours’ exposure, Boscalid and Bixafen (1 µM) induced a significant increase in mitochondrial superoxide production, multiplying its level by 8-fold and 12-fold, respectively, in HepG2 cells compared with untreated cells (Figure 5). Of note, the level of global ROS was unchanged in HepG2 cells as revealed by the H_2_DCFDA assay (Figure 6). In PBMCs, the level of mitochondrial superoxide was not significantly changed after 2 hours’ SDHI exposure. In BJ-fibroblasts, Bixafen treatment increased the superoxide production, while Boscalid did not. Of note, the level observed after Bixafen treatment in BJ was much lower than that achieved in HepG2 cells.

### 2.3. Bixafen and Boscalid Induced Apoptosis in HepG2 Cells, but Not in PBMC and BJ Cells

**Figure 7:**
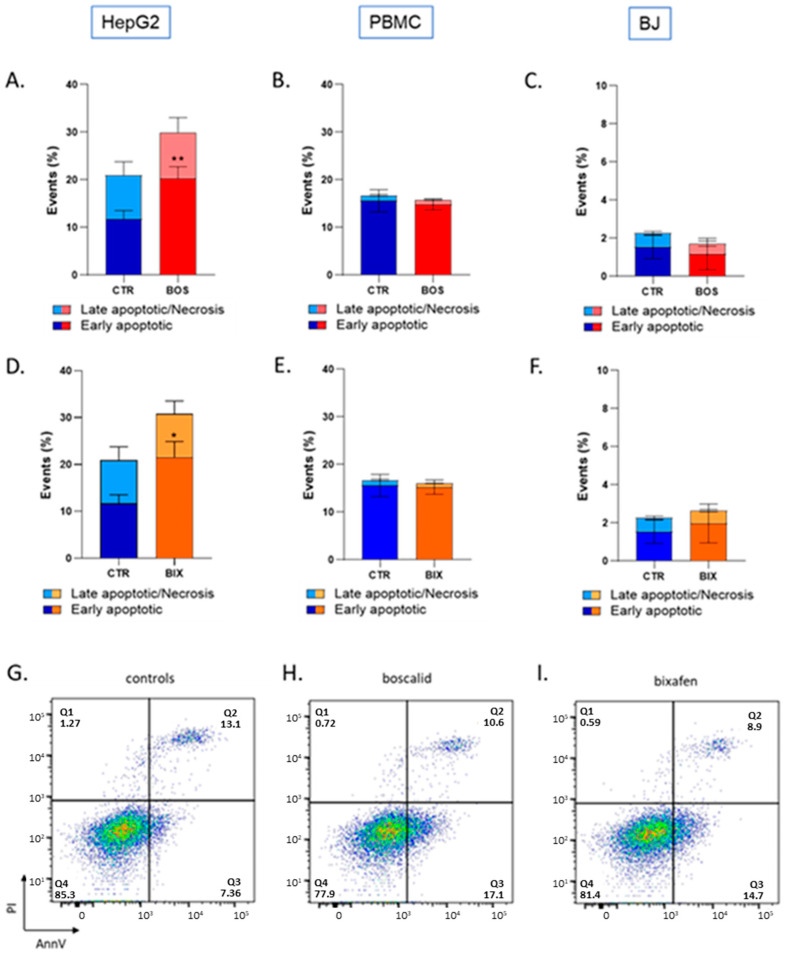
Effect of SDHI exposure on cytotoxicity. Top and middle rows: histograms of percentage of early apoptotic cells/late apoptotic or necrotic cells in control (DMSO 0.1%) and SDHIs-exposed human cells (1 µM for 2 h). Top row: Boscalid-treated cells. (**A**): HepG2 liver cells, (**): *p* < 0.01. (**B**): PBMCs, (**C**): BJ-fibroblasts. Middle row: Bixafen-treated cells. (**D**): HepG2 liver cells, (**E**): PBMCs, (**F**): BJ-fibroblasts. Bars represent mean ± SEM (% events). N = 3, (*): *p* < 0.05. Bottom row: Representative Annexin V/PI flow cytometry plots of HepG2 cells. (**G**): control cells, (**H**): Boscalid-treated cells, (**I**): Bixafen-treated cells.

By altering mitochondrial function, we hypothesized that SDHIs could induce apoptosis. Flow cytometry was used to assess early apoptotic and late apoptotic/necrotic cells after SDHI exposure. A significant increase in early apoptotic cells, but not in late apoptotic/necrotic cells, was observed in HepG2 cells after 2 hours’ exposure to Boscalid and Bixafen (Figure 7). In PBMC and BJ cells, no significant increase in cell mortality was found after short-term exposure to Boscalid and Bixafen (Figure 7).

## 3. Discussion

In this study, we found that short-term exposure (2 h) of three human cell lines to SDHI-fungicides Boscalid and Bixafen (1 µM) led to a mitochondrial dysfunction as the oxygen consumption was significantly reduced (Figure 2). Our results on the mitochondrial function are consistent with a previous study where enzymatic assays showed that SDHI-fungicides inhibited the human SDH with IC50 in the µM range [4]. We confirm here that SDHI-fungicides (Boscalid and Bixafen) are not selectively poisoning fungi, but are also active on non-target human cells. Despite this alteration in cell respiration, we did not observe a significant change in ATP level (Figure 3) after 2 hours’ and 24 hours’ exposure, suggesting that compensatory mechanisms may exist to keep cellular bioenergetics.

An important consequence of the inhibition of the ETC in human cells was the increase in mitochondrial superoxide level in HepG2 cells. In BJ-fibroblasts, only Bixafen induced an increase in mitochondrial superoxide production (Figure 5). The fact that BJ-fibroblasts display high antioxidant capacity [30] may likely explain the discrepancy between the effect on OCR and the absence of effect on mitochondrial superoxide production in this cell line. HepG2 cells were the most responsive to both SDHIs, with an 8-fold and 12-fold increase in superoxide after Boscalid and Bixafen treatment, respectively. These results agree with the important decrease in OCR observed in those cells after SDHI exposure and could be explained by the intrinsic feature of hepatocytes that contain more mitochondria compared with other cell types. Of note, as shown in Figure 2, basal OCR measured in HepG2 cells was much higher than in other cells analyzed in the present study. Regarding mitoROS production, Bixafen displayed more marked results than Boscalid (Figure 5). This could be explained by the fact that new generation SDHIs such as Bixafen possess a methyl-pyrazol fragment capable of inhibiting the ubiquinol cytochrome c reductase (complex III) of ETC, in addition to the inhibition of SDH (complex II) (Figure 1) [4]. Interestingly, the global level of ROS (as measured by the H_2_DCFDA assay) after exposure of HepG2 cells was not significantly modified (Figure 5) while the level of mitochondrial superoxide was dramatically increased, suggesting that the higher ROS production seems confined to the mitochondrial compartment. A similar observation of selective mitochondrial superoxide production was previously reported in superinvasive cancer cells [27]. Of note, global ROS production was observed in other studies, but after longer exposure and higher concentrations of Boscalid in zebrafish (14.5 µM, 6 to 18 h post-fertilization) [6] and in *Chlorella vulgaris* (4.5 µM, 96 h) [31]. An increase in SOD activity was also reported in human fibroblasts (from a patient with familial Alzheimer disease) exposed during 13 days to 1 µM Bixafen [4]. In the future, it would be interesting to investigate the nature of ROS produced using other methods, for example, those using fluorescent probes coupled to HPLC [32,33,34].

We further explored if the increase in mitochondrial ROS could lead to the induction of apoptosis. By altering mitochondrial function, SDHIs could be capable of releasing cytochrome c and initiating the apoptotic cascade. Here also, HepG2 cells demonstrated a high susceptibility to SDHI exposure, as we observed an increase in early apoptotic cells after both Boscalid and Bixafen (1 µM) short-term exposure (Figure 7). No significant induction of apoptosis was observed in PBMCs and in BJ-fibroblasts (Figure 7). The latter results are the likely consequence of the smaller increase in mitochondrial ROS production (Figure 5) in those cells lines compared with HepG2 cells.

Overall, our results demonstrated that a short-term exposure to Boscalid and Bixafen leads to mitochondrial dysfunction in human cells. While bioenergetics was not affected, the SDHI exposure led to an increase in mitochondrial ROS in cells with insufficient antioxidant capacity and to an induction of apoptosis in cells with the highest production of mitochondrial superoxide.

As mentioned earlier, the cell lines used in the present study are representative of cells that are most likely to be in contact with SDHIs after exposure (blood, liver and skin). Of note, the HepG2 cell is not a normal hepatocyte but derives from a liver biopsy of a differentiated hepatocellular carcinoma. However, the HepG2 human cell line is a model classically used by researchers and pharmaceutical companies to assess the hepatoxicity of chemicals or drugs [35,36,37,38]. In the future, it would be interesting to enlarge the effect of SDI-fungicides to other cell lines as SDH deficiency has been shown to be associated with encephalopathy and cardiomyopathy [39]. In addition, it would be interesting to assess the effect of repeated short-term exposure to SDHI-fungicides on human cells.

At this stage, it is premature to extrapolate these results to human health after SDHI exposure. As mentioned above, the concentration of 1 µM is likely close to concentrations achieved after exposure to levels close to the AOELs for operators. There is a large uncertainty regarding the real concentrations achieved in the blood of people potentially exposed to SDHI-fungicides. Many parameters such as frequency of spreading, exposure periods, amounts of SDHIs manipulated, protection means, and contaminated food diets may strongly influence the level of SDHIs that will be achieved in tissues. As far as we know, there are no epidemiologic data existing about blood concentration levels after exposure in humans. The exposure to pesticides in farmers and the general population living close to a treated area is mostly evaluated through quantification of pesticides in hair [40,41]. Our results call for such biomonitoring to evaluate the real level of exposure and to drive future research with a range of concentrations established experimentally rather than theoretically. Moreover, a reevaluation of AOELs/ADIs for SDHI-fungicides could be debated. AOELs/ADIs are established by extrapolation of toxicology data obtained in animals and by defining a theoretical security factor. Accounting for experimental dose/toxicity relationships in human cells could be desirable.

## 4. Materials and Methods

### 4.1. Reagents

**Figure 8:**
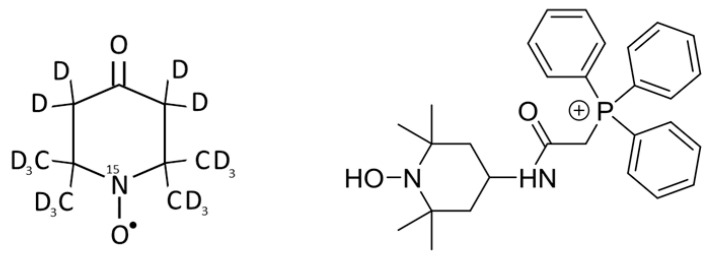
Chemical structures of ^15^N-PDT used as a sensor for oximetry (**left**) and Mito-TEMPO-H used as a sensor for mitochondrial superoxide production (**right**).

^15^N-PDT (4-oxo-2,2,6,6-tetramethylpiperidine-d_16_-^15^N-1-oxyl) (Figure 8) originated from CDN Isotopes (Pointe-Claire, Canada). Mito-TEMPO-H (2-(2,2,6,6-Tetramethylpiperidin-1-oxyl-4-ylamino)-2-oxoethyl triphenylphosphonium chloride) (Figure 7) was purchased from Enzo Lifescience (Antwerpen, Belgium). Boscalid (2-chloro-N-(4-chlorobiphenyl-2-yl)nicotinamide), Bixafen (N-[2-(3,4-dichlorophenyl)-4-fluorophenyl]-3-(difluoromethyl)-1-methylpyrazole-4-carboxamide), superoxide dismutase 2 conjugated with polyethylene glycol (PEGSOD2), ATP disodium, diethylenetriaminepentaacetic acid (DTPA), dextran from *Leuconostoc Mesenteroides* (average MW 60000–76000) and dimethyl sulfoxide (DMSO) were from Sigma-Aldrich (Overijse, Belgium). The cell-permeant 2′,7′-dichlorodihydrofluorescein diacetate (H_2_DCFDA) came from Invitrogen^TM^ (Waltham, MA, USA).

### 4.2. Cell Lines and Culture

All cell lines were purchased from The American Type Culture Collection (Manassas, VA, USA) and maintained in a humidified atmosphere with 5% CO_2_ at 37 °C. HepG2 (human hepatocarcinoma) and BJ (human skin fibroblasts) cells were routinely cultured in Eagle Minimum Essential Medium supplemented with 10% heat-inactivated fetal bovine serum (FBS) (Thermo Fisher Scientific). Hs27 (human skin fibroblasts) and PBMC (peripheral blood mononuclear cells) were cultured in Dulbecco’s Modified Eagle’s Medium (DMEM) and RPMI-1640 respectively, both supplemented with 10% of FBS.

### 4.3. EPR Oximetry

EPR oximetry is based on the use of an oxygen-sensing probe, ^15^N-PDT (4-oxo-2,2,6,6-tetramethylpiperidine-d_16_-^15^N-1-oxyl), to measure variations in oxygen levels in samples, and subsequently cells’ oxygen consumption rate (OCR) in a sealed capillary. ^15^N-PDT EPR linewidth is very narrow due to deuterium substituting hydrogen, providing a high sensitivity to measure the broadening induced by the oxygen content in the preparation. Oxygen consumption rate (OCR) of cell lines was measured using a Bruker EMX-Plus spectrometer operating in X-band (9.85 GHz) and equipped with a PremiumX ultra low noise microwave bridge and a SHQ high sensitivity resonator. The EPR cavity was heated at 310 K with continuous nitrogen flow during all experiments. Respiration mixture was composed of 60 µL of previously harvested cells (stock solution 5.10^6^ cells/mL of culture medium), 40 µL of Dextran solution (20%) and 4 µL of ^15^N-PDT (2 mM), transferred into a hematocrit capillary sealed with gum. The final device was inserted into a quartz tube and put into the EPR cavity. Experimental parameters for acquisition set in the Bruker Xenon Spin fit program were: microwave power, 2.518 mW; modulation frequency, 100 kHz; modulation amplitude, 0.005 mT; center field, 335 mT; sweep width, 1.5 mT; sweep time, 15 s. An automated “2D-field-Delay” measurement was launched 3 min after probe mixing, counting 15 points with a time delay of 60,000 ms. Data analysis was performed switching to processing mode and using “peak picking” on selected regions of the ^15^N-PDT –peaks. The final file was saved as an ASCII file to extract linewidth data at each point. ^15^N-PDT linewidth was correlated with the % of oxygen with a calibration curve. OCR corresponded to the slope of oxygen level during time.

### 4.4. EPR Superoxide Measurement

Mitochondrial superoxide measurements by EPR were based on the use of Mito-TEMPO-H, a cyclic hydroxylamine, able to detect superoxide in complex biological media with high sensitivity. Thanks to its tryphenylphosphonium moiety (TPP^+^), this probe enters into and accumulates within mitochondria, enabling specific measurement of mitochondrial superoxide. Superoxide production was monitored using a Bruker EMX-Plus spectrometer operating in X-band (9.85 GHz) and equipped with a PremiumX ultra low noise microwave bridge and a SHQ high sensitivity resonator. The EPR cavity was heated at 310 K with continuous air flow during all experiments. The mixture combined 37 µL of cell suspension (stock solution: 20.10^6^ cells/mL culture medium), 0.56 µL of DTPA (100 mM), 5 µL of PBS ((1×) − pH 7.4) and 7.5 µL of Mito-TEMPO-H (1mM). Superoxide production contribution was assessed by making another measurement using the same conditions but adding 2.5 µL of PEG-SOD2 (4000 U/mL) to replace 2.5 µL of PBS. PEG-SOD2 was incubated 15 min before adding the Mito-TEMPO-H probe. Previous studies have shown that the pre-incubation of cells in the presence of PEG-SOD allows the cellular uptake of the enzyme and intracellular scavenging of superoxide [42]. Mito-TEMPO-H stock solution was flushed with Argon prior and during pipetting to avoid probe oxidation. The final solution was transferred into a PTFA tube (inside diameter 0.025 in., wall thickness 0.002 in.) cutting of 12 cm using a needle and folded in 6 before being inserted into an open quartz tube. Experimental parameters for acquisition set in the Bruker Xenon Spin fit program were: microwave power, 20 mW; modulation frequency, 100 kHz; modulation amplitude, 0.1 mT; center field, 336.5 mT; sweep width, 1.5 mT; sweep time, 30.48 s. An automated “2D-field-Delay” measurement was launched 3 min after probe mixing, counting 11 points with a time delay of 40,000 ms. Data analysis was performed switching to processing mode and using “Integration & derivative” and “double integration” on selected regions of the peaks. The final file was saved as an ASCII file to extract double integration (DI) data at each timepoint. Point 1 DI was subtracted from point 11 DI for each condition. Superoxide contribution was measured by subtracting mean PEGSOD2 DI to mean control DI.

### 4.5. Cell Death Detection by Flow Cytometry

Apoptotic changes in cells were assessed using the eBioscience™ Annexin V Apoptosis Detection Kit APC (Thermofisher, Waltham, MA, USA). A total of 50,000 cells per condition were plated 2 days before treatment in T25 flasks. On the day of the experiment, cells were treated for 2 h with 1 µM of Boscalid, Bixafen or control solution (DMSO 0.1%) mixed into their respective culture media. Cells were then detached with trypsin (0.25% trypsin-EDTA was used for HepG2 cells), harvested into their respective old media, centrifuged and transferred into a FACS tube with fresh media. The staining protocol briefly included washing the cells with PBS, then adding 80 µL of binding buffer along with 5 µL Annexin V (AnnV) and 5 µL Propidium Iodide (PI) per tube. Cells were kept in the dark for 15 min until FACS assessment, with an additional 100 µL of binding buffer. Data were acquired using a BD FACSCantoII flow cytometer equipped with Diva Software and processed with FlowJo software. At least 10,000 events were analyzed for each condition. Cells’ subpopulations were identified according to unstained control cells and distinguished between alive (AnV− PI−), early apoptotic (AnV+ PI−) and late apoptotic/necrotic (AnV+ PI+).

### 4.6. Global ROS Production Using H_2_DCFDA

Intracellular ROS were detected by flow cytometry using a cell-permanent 2′,7′-dichlorofluorescein diacetate (H_2_DCFDA) fluorescent probe. Once inside the cell, the probe was cleaved by endogenous esterases and became fluorescent after oxidation by ROS. HepG2 cells were treated for 2 h with 1 µM of Boscalid (BOS) and Bixafen (BIX), or control solution (DMSO 0.1%) then incubated with H_2_DFCDA (10 µM final concentration) for 30 min at 37 °C. Cells were then harvested with 0.25% trypsin-EDTA solution, suspended into fresh medium, and immediately analyzed by flow cytometry. Data were acquired using a FACSCanto II cytofluorimeter equipped with Diva Software and processed with FlowJo software.

### 4.7. Cellular ATP Production

HepG2 ATP production was measured using the CellTiter-Glo^®^ Luminescent Cell Viability Assay (Promega, Madison, WI, USA). HepG2 were cultured on a 96-well plate then treated the day after with 1 µM of Boscalid (BOS) and Bixafen (BIX), or control solution (DMSO 0.1%) for 2 and 24 h (100 µL media/well). ATP quantification was performed with a standard curve using ATP disodium (10 nM to 1 µM) in cell-free media. The entire plate was then mixed for 2 min on an orbital plate after 100 µL of reagent was added to each well. Luminescence was measured with the SpectraMax at 560 nm after 10 min incubation at RT to stabilize signal.

### 4.8. Statistics

Data were represented as means ± SEM. All experiments were performed in triplicates or more. Student T tests were applied for OCR and superoxide measurements experiments, whereas One-way ANOVA were performed on all other experiments.

## 5. Conclusions

A short-term exposure of the HepG2 human cell line to SDHI-fungicides Boscalid and Bixafen induces a mitochondrial dysfunction, altering the oxygen consumption, the mitochondrial ROS production, and eventually leading to apoptosis. Further research is warranted to assess the effect of repeated exposures and relevance of these observations for human health.

## Data Availability

Available on request.
